# Prevention of Recurrent Benign Paroxysmal Positional Vertigo: The Role of Combined Supplementation with Vitamin D and Antioxidants

**DOI:** 10.3390/audiolres12040045

**Published:** 2022-08-22

**Authors:** Giacinto Asprella Libonati, Antonello Leone, Salvatore Martellucci, Andrea Gallo, Roberto Albera, Sergio Lucisano, Maurizio Bavazzano, Giuseppe Chiarella, Pasquale Viola, Francesco Galletti, Francesco Freni, Francesco Ciodaro, Vincenzo Marcelli, Giuseppe Tortoriello, Leonardo Scotto di Santillo, Pasqualina Maria Picciotti, Jacopo Galli, Silvano Vitale, Nicola Quaranta, Giada Cavallaro, Paolo Gamba, Roberto Teggi, Iacopo Cangiano, Mario Faralli, Annalisa Barboni, Aldo Messina, Giusi Graziano

**Affiliations:** 1 Vestibology & ENT Unit, Giovanni Paolo II Hospital, 07026 Policoro, Italy; 2 Otorhinolaryngology Unit, Santa Maria Goretti Hospital, AUSL, 04100 Latina, Italy; 3 Department of Sense Organs, Sapienza University of Rome, 00185 Rome, Italy; 4 Department of Surgical Sciences, University of Turin, 10124 Turin, Italy; 5 Department of Otorhinolaryngology, Head and Neck Surgery, Clinical and Instrumental Vestibology, San Martino Polyclinic of Genova, 16132 Genoa, Italy; 6 Audiology Unit, Regional Centre of Cochlear Implants and ENT Diseases, Department of Experimental and Clinical Medicine, Magna Graecia University, 88100 Catanzaro, Italy; 7 Otorhinolaryngology Unit, Department of Adult and Development Age Human Pathology “Gaetano Barresi”, Policlinico G. Martino, University of Messina, 98122 Messina, Italy; 8 Otorhinolaryngology Unit, Ospedale del Mare, 80147 Naples, Italy; 9 Vestibology and Vestibular Rehabilitation Service, ASL 3 Genovese, 16162 Genoa, Italy; 10 Otolaryngology Institute, Department of Head and Neck, Fondazione Policlinico Universitario A. Gemelli IRCCS, Università Cattolica del Sacro Cuore, 00168 Rome, Italy; 11 Phoniatrics Unit ASL Lecce, Department of Rehabilitation, 73100 Lecce, Italy; 12 Otolaryngology Unit, Department of Basic Medical Sciences, Neuroscience, and Sense Organs, University of Bari “Aldo Moro”, 70121 Bari, Italy; 13 Department of Otorhinolaryngology, Head and Neck Surgery, Laboratory of Clinical and Instrumental Vestibology-Poliambulanza Foundation Hospital, 25124 Brescia, Italy; 14 ENT Division, San Raffaele Scientific Institute, 20132 Milano, Italy; 15 Section of Otolaryngology-Head and Neck Surgery, Department of Surgical and Biomedical Sciences, University of Perugia, 06122 Perugia, Italy; 16 Otoneurology and Tinnitus Unit, Chair of Audiology, P. Giaccone University Hospital, 90127 Palermo, Italy; 17 Center for Outcomes Rsearch and Clinical Epidemiology (CORESEARCH), 65100 Pescara, Italy

**Keywords:** vitamin D, BPPV, high recurrence BPPV, BPPV relapses, LICA^®^, antioxidants, otoconia, otoliths

## Abstract

Benign paroxysmal positional vertigo (BPPV) usually has a favorable course, although it is possible to observe BPPV with a high recurrence rate. Previous studies suggested that vitamin D deficiency might affect BPPV recurrences, and oxidative stress might play a complementary role in BPPV pathogenesis. This multicentric trial aimed to evaluate the effectiveness of oral nutritional supplementation with a compound of alpha-lipoic acid, Carnosine, and Zinc (LICA^®^ (Difass International, Coriano (RN), Italy)), vitamins of group B and vitamin D in preventing BPPV recurrences. A total of 128 patients with high recurrence-BPPV were randomized in three arms: Arm 1 consisted of subjects with “insufficient” or “deficient” vitamin D blood levels, treated with daily oral supplementation of LICA^®^, vitamins of group B and vitamin D3 (800 UI), Arm 2 included BPPV subjects with “sufficient” vitamin D who did not receive any nutritional support, and Arm 3 included subjects with a “sufficient” serum concentration of vitamin D who received supplementation with a compound of LICA^®^ and Curcumin. After six months of follow-up, a significant reduction of BPPV relapses compared to the baseline was found only in Arm 1 (−2.32, 95% CI: 3.41–1.62, *p*-value < 0.0001). Study results suggested that oral nutritional supplementation with vitamin D3 plus antioxidants can prevent relapses in patients suffering from high recurrence-BPPV.

## 1. Introduction

Benign paroxysmal positional vertigo (BPPV) is characterized by recurrent vertigo spells triggered by head movements, and it is considered the most common peripheral vestibular disorder [1,2].

According to the most accredited theory, BPPV is an otoconia-linked disorder due to otoconial debris detached from the utricular macula and displaced within the semicircular canals. The most common pathophysiological mechanisms proposed for BPPV are canalolithiasis and cupulolithiasis. In the first one, the free-floating otolithic mass gravitates along the canal lumen as a consequence of the position assumed by the head with respect to the axis of gravity; in doing so, it pushes the endolymphatic column like a leaky piston [3], leading to a transient cupular deflection. The cupulolithiasis theory claims that otoliths are attached to the ampullary cupula surface, thus making it heavier and gravity sensitive [4,5].

BPPV can be classified according to the affected semicircular canal (SC) and the involved arm, as follows [1,2,3,4,5,6,7,8]:

- Posterior Semicircular Canal (PSC) BPPV: Geotropic and Apogeotropic variant
- Horizontal Semicircular Canal (HSC) BPPV: Geotropic and Apogeotropic variant
- Anterior Semicircular Canal (ASC) BPPV
- Multicanal BPPV

The involvement of a single semicircular canal represents the most frequent condition, although BPPV might simultaneously affect more semicircular canals on one or both sides. PSC BPPV is the most frequent variant (75–80%), followed by HSC BPPV [1,2,3,4,5,6,7,8].

The diagnosis of the involved canal and its variant, apogeotropic or geotropic, is based on the detection of the typical positional paroxysmal nystagmus induced by performing the diagnostic maneuvers and should always be based on the monitoring step-by-step of the evoked nystagmus [8,9]. A nystagmus-based approach strategy allows us to perform the repositioning maneuvers by monitoring in real-time the movement of the canaliths within the involved canal and in each of its arms [1,3,4]. However, BPPV can be a severely disabling condition due to either an inadequate response to physical therapy or a high recurrence rate, both resulting in a considerable individual and socio-economic burden [1,2,3,4,5,6,7].

Although BPPV is considered idiopathic in nearly 80% of cases, in selected cases, it might be a consequence of either triggering events or general conditions inducing otoconial detachment, such as head trauma, recent ear surgery, dental implant, inner ear disorders, Menière disease, and migraine. Furthermore, although the natural history of idiopathic BPPV usually has a favorable course, including spontaneous remissions within days or weeks, it is possible to observe BPPV with a high recurrence rate, with a lifetime relapse of 2.4% [1,2,3,4].

These data stimulated a growing interest of researchers in the otoconial processes of formation, degradation, and disposal: it is currently known that otoconia are prone to damage by drugs intake, inflammation, trauma, and decalcification due to elder age; this last one induces a progressive demineralization leading to degradation and fragmentation of otoconia and, consequently, to balance disorders [10,11].

Otoconia in mammals’ inner ears are composed of calcium carbonate crystals and glycoproteins. In recent years, several studies suggested that calcium metabolism disorders related to vitamin D deficiency might affect otoconia homeostasis, increasing the BPPV recurrence and the incidence of complicated forms [12,13,14,15,16,17]. An increasing number of studies investigated the efficacy of 1,25-dihydroxyvitamin D (or vitamin D3) supplementation in reducing BPPV recurrence [13,18,19,20,21].

Calcium metabolism also appears closely linked with oxidative stress. The endoplasmic reticulum, the cellular organelle responsible for calcium storage, can increase the influx of calcium under stress conditions, which in turn triggers the cascade reaction of reactive oxygen species (ROS) formation in mitochondria. Furthermore, some findings suggested that oxidative stress may have a synergistic or complementary role in the pathogenesis of BPPV [22,23].

This work aims to evaluate the effectiveness of oral nutritional supplementation with a compound of alpha-lipoic acid (ALA), L-carnosine and zinc (LICA^®^), vitamins of group B and vitamin D3 in preventing recurrence of BPPV over six months follow up. Other secondary endpoints are to evaluate the incidence of vitamin D deficiency in patients diagnosed with recurrent BPPV and to assess the post-maneuver residual dizziness and its improvement correlated to the correction of oxidative stress with LICA^®^ in patients with and without vitamin D deficiency.

## 2. Materials and Methods

Fifteen referral centers conducted this interventional multicenter randomized 3-arm clinical trial after the approval of the Institutional Review Board. Patient enrollment and data collection were conducted from January 2017 to June 2020.

The study included patients of both sexes, aged between 18 and 85 years and diagnosed with unilateral or bilateral idiopathic BPPV with high recurrence rate, involving the posterior semicircular canal or the lateral semicircular canal (both in geotropic and apogeotropic form). The BPPV recurrence rate was evaluated according to the following enrollment criterion: patients with two or more episodes of BPPV within the previous six months or with at least three episodes in the previous 12 months, with a symptom-free interval of at least 3 weeks between two consecutive episodes.

Patients under 18 y.o., pregnant or lactating women, and patients with vitamin D values over 100 ng/mL (>250 nmol/L) were not eligible for the study. Secondary BPPV was considered as an additional exclusion criterion. Patients with conditions associated with high BPPV recurrence or massive otolithic detachment, such as craniofacial surgery in the previous three months, head injury within the last 30 days, and clinical history of otoneurological pathology other than BPPV (migraine, vestibular neuritis, Menière’s disease) were excluded from the study.

All enrolled patients were subjected to evaluation of positional nystagmus by performing the diagnostic tests commonly used for each semicircular canal under monitoring with video-Frenzel goggles.

### 2.1. Study Design

The enrolled patients were divided into the following three arms:

- Arm 1: Including patients with “insufficient” (between 20 and 30 ng/mL) or “deficient” (less than 20 ng/mL) serum concentration of 25-hydroxyvitamin D or 25 (OH) D who were given food supplementation with LICA^®^, vitamin D3, and vitamins of group B composed as follows: LICA^®^ (ALA 600 mg, L-carnosine 165 mg, zinc 7.5 mg), vitamin B2 0.8 mg, vitamin B6 1 mg, vitamin D3 800 UI (Vertistop^®^ D (Difass International, Coriano (RN), Italy), 1 tablet/day before meals).
- Arm 2: Including patients with “sufficient” serum concentration of 25 (OH) D (between 31 and 100 ng/mL) and no treated
- Arm 3: Including patients with “sufficient” serum level of 25 (OH) D (between 31 and 100 ng/mL) who were given food supplementation with LICA^®^ and Curcumin composed as follows: LICA^®^ (ALA 600 mg, L-carnosine 165 mg, zinc 7.5 mg), Curcumin 100 mg, Piperine 1 mg (Vertistop^®^ L, 2 tablets/day, b.i.d. before meals).

The trial was organized according to the following study design in 5 visits over six months, with four subsequent 25 (OH) D serum levels assessments. During the enrollment visit (Visit 0), BPPV was diagnosed, searching for the typical nystagmus evoked by the positioning diagnostic tests, and treated with liberatory techniques suitable for each BPPV variant. Then, the criteria for inclusion in the study were verified. The randomization visit (Visit 1) was performed 3–7 days after Visit 0. After verifying the previously diagnosed BPPV resolution, patients were assigned to Arm 1 in case of serum vitamin D3 values below 30 ng/mL and randomly assigned to Arm 2 or 3 in case of serum vitamin D values above 30 ng/mL. During the following three bimonthly visits (Visit 2–4), the occurrence of any relapses in the previous two months was assessed, and in case of compensation of the vitamin D3 deficit, the patients were randomly assigned to Arm 2 or Arm 3; conversely, subjects who found lowering of the serum 25 (OH) D concentration, below the “sufficiency” threshold, switched in Arm 1.

Patients were asked to contact the referral center in case of relapse of symptoms during the follow-up. In this case, the patient was re-evaluated within 48/72 h after symptoms onset, and if the diagnostic tests were positive, they were subjected to a new suitable treatment with liberatory maneuvers. The number of relapses at the end of the six months of study (186 ± 5 days) and the total number of maneuvers necessary to resolve each episode of BPPV was recorded. A qualitative and quantitative assessment of post-maneuver residual dizziness was also carried out for each treatment. The details of the diagnostic protocol are listed below.

### 2.2. Diagnostic Protocol and Vestibular Symptoms Assessment

All patients with clinical history of positional vertigo underwent a bedside neurotologic evaluation, including examinations for ocular alignment, spontaneous and gaze-evoked nystagmus, vestibulo-ocular reflex, saccades, smooth pursuit, and diagnostic positional tests for BPPV assessment. BPPV assessment was achieved using Upright BPPV Protocol [8,24,25,26] and/or using traditional positioning test: Semont diagnostic maneuver and Dix-Hallpike Test for PSC BPPV and Supine Head Yaw Test (also known as Pagnini-McLure or Supine Head Roll Test) for HSC BPPV [1].

Once the diagnosis of BPPV was reached, and other vestibular pathologies were excluded, patients were treated with the appropriate physical treatment, according to the involved semicircular canal. PSC BPPV was treated using Epley canalith repositioning procedure or Semont therapeutic maneuver, whereas HSC BPPV was managed with Gufoni maneuver or barbecue-roll techniques [1,2,3,4,5].

All the enrolled patients were asked for the following clinical additional information: sun exposure (high, moderate, absent), intake of milk and dairy products in the diet, diagnosis of osteoporosis, and implementation of vitamin D plus calcium, and concomitant pathologies.

The Italian validated version of Dizziness Handicap Inventory (DHI) [27] was submitted to all patients at the diagnosis and at every follow-up visit with VNS (Verbal Numerical Scale), and VAS (Visual Analogue Scale) scales to evaluate positional vertigo and residual dizziness (RD).

### 2.3. Statistical Analysis

Continuous variables were expressed as mean ± standard deviation, categorical ones as absolute numbers and proportions. The clinical and demographic features of patients were compared in the three treatment groups through the Kruskal–Wallis and Chi-square test, based on the nature of the variables. In many cases, it was appropriate to use Fisher’s exact test. The Spearman’s rank coefficient of correlation was instead calculated to verify linear correlations between scales. Linear and generalized linear mixed effects models for repeated measures were used to compare in the three treatment groups the variation of the number of relapses (primary outcome) and of the values of evaluation scales (secondary outcomes) between the baseline visit and the last clinical visit of the study. The results were expressed as estimated means, estimated mean differences from baseline visit, and relative confidence intervals at 95%. Multivariate analyzes were conducted in order to evaluate the same outcomes in case of confusing variables. The choice of factors to be included in the models was guided by statistical and clinical criteria. The different outcomes were subjected both to Intention to Treat (ITT) and Per Protocol (PP) analysis.

For all statistical analyses, *p* value < 0.05 was considered significant, and it was used SAS software (version 9.4) (SAS Institute Inc., Cary, NC, USA).

## 3. Results

A total of 128 patients were enrolled in the study. A different distribution of subjects was observed across the three arms of the trial: based on serum 25 (OH) D values at the time of the randomization visit, 93 patients (72.65%) were assigned to Arm 1. Among the three groups, however, no statistically significant differences were observed concerning age, sex, number of relapses, and baseline DHI, VNS, and VAS values (*p* > 0.05). Statistically significant differences were instead observed in the prevalence of osteoporosis. Demographic characteristics are shown in Table 1.

The distribution of patients across the three treatment arms varied during the study according to treatment dropouts and switches determined by changes in serum concentration of 25 (OH) D recorded during follow-up visits. Table 2 reported the distribution of patients at randomization, after each follow-up visit, and at the end of the trial.

**Figure 1:**
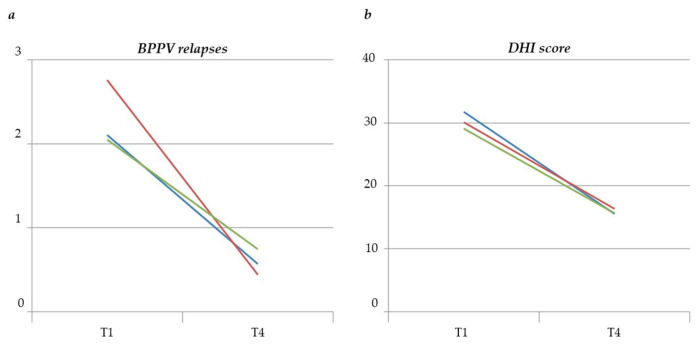
Variation of BPPV relapses (**a**) and DHI score (**b**) from randomization visit (T1) to the end of the study (T4). Red line: Arm 1, blue line: Arm 2, green line: Arm 3.

At the end of the study, the ITT multivariate mixed model analysis showed a statistically significant reduction in the number of BPPV relapses (−2.32, 95% CI: 3.41–1.62, *p*-value < 0.0001) in Arm 1 patients. No statistically significant changes in the number of relapses were found in the other two treatment groups (Figure 1a).

In Arm 1, an improvement was also found in all subjective assessment parameters, as shown in Table 3. All three treatment arms recorded a statistically significant reduction in the mean DHI score (Figure 1b).

Results of the ITT multivariate mixed model analysis are reassumed in Table 3.

## 4. Discussion

The vitamin D status depends on the production of vitamin D3 in the skin under the influence of ultraviolet radiation and vitamin D intake through the diet or vitamin D3 supplements. The production of vitamin D3 in the skin depends on sunshine exposure, latitude, and skin pigmentation, and 25 (OH) D is lower with higher latitudes and darker skin types, although there are some exceptions. Hypovitaminosis D is a widespread condition in the general population, and its prevalence is influenced by age and sex [28,29,30]. A large-scale study in the United Kingdom, including subjects from 18 to 85 y.o. and over, involving institutionalized people, revealed 15–25% of vitamin D deficiency (25 (OH) D serum concentration <25 nmol/L) in adolescents and young subjects and over 35% in people institutionalized and/or over 85 years [31]. Many studies have been conducted on specific subpopulations considered at risk. Vitamin D status was studied in 700 women between 60 and 80 years old in the Italian population: mean serum 25 (OH) D was 27 nmol/L, and serum 25 (OH) D level was below 25 nmol/l in more than 50% [32].

In recent literature, serum 25 (OH) D levels were correlated with higher BPPV occurrence, although some Authors have provided controversial results [12,13,14,15,16,17,18,19,20,21,33].

One of the endpoints of our study was to evaluate the incidence of vitamin D deficiency in patients with high-recurrences BPPV enrolled in fifteen Italian centers. Our results confirmed that the prevalence of hypovitaminosis D in those patients was significantly higher compared to the general population and that the serum concentration of 25 (OH) D was lower than in populations considered at risk, such as women over 60 y.o. Our study, confirming the observation reported by a study performed on the north Sardinia population [34], indicates that patients with complicated BPPV should be considered patients at high risk of hypovitaminosis D and suggests the opportunity to perform the vitamin D assessment routinely in patients with relapsed BPPV in addition to the standard treatment with repositioning maneuvers.

It has long been known otoconia are generated through a dynamic calcium uptake: such e process requires maintaining calcium concentration locally high to promote the crystallization of the otoconia and low in the rest of the endolymph to promote the reabsorption of detached otoconia [35,36]. A complex system of calcium-binding proteins and an epithelial calcium transport mechanism are involved in inner ear calcium homeostasis with apical entry channels (TRPV5 and TRPV6), proteins binding cytoplasmic calcium (cal-bindin-D9K and calbindin-D28K) and pump extruding calcium from the basolateral membrane (sodium-calcium and calcium-ATPase exchangers). Such a calcium transport system is also present in the membranous labyrinth, and vitamin D is active in up-regulating it [37,38]. Vitamin D deficiency determines a down-regulation of the calcium-binding protein system and the epithelial calcium transport system, and consequently, a defect in otoconia production (“otolithoporosis”) with increased otolithic detachment and an increase in calcium concentration free in the rest of the endolymph with a reduced reawakening of free otoliths in the endolymph [39,40].

This evidence and the outcomes of some recent trials [17,18,19,20,41] and meta-analysis [22,42] support the therapeutic effectiveness of the correction of hypovitaminosis D in patients with recurrent BPPV.

In our study, patients with serum concentration of 25 (OH) D < 30 ng/mL were assigned to Arm 1 and subjected to food supplementation with antioxidants (alpha-lipoic acid, Carnosine, and Zinc, a patented compound known as LICA^®^), vitamin D3 (800 IU), and vitamins of group B.

A statistically significant reduction in the number of BPPV relapses was observed in this group of patients. These data suggested that patients with high recurrence of BPPV and hypovitaminosis D may have an altered calcium metabolism in the inner ear.

Therefore, oral nutritional supplementation with vitamin D3 probably promotes a positive effect in the prevention of labyrintholithiasis by exerting a direct action on calcium metabolism in the inner ear, which improves otolith compactness, reducing their detachment. However, it is likely that vitamin D3 also exerts an “indirect” effect by modulating oxidative stress in the inner ear, improving microcirculation. The administration of vitamin D in association with a powerful antioxidant (LICA^®^) enhances its effects resulting in an improvement not only in labyrintholithiasis but also in the recovery times of the post-maneuver residual dizziness.

Vitamin D3 regulates the expression of numerous target genes via the nuclear vitamin D receptor (VDR), which controls downstream events, including the protective role against oxidative stress, regulation of autophagic pathways, and the interaction between apoptosis and cell survival pathways. Vitamin D deficiency promotes oxidative damage and increases cell apoptosis through various mechanisms: increased production of oxygen free radicals, increased expression of apoptosis markers, and alteration of mitochondrial function. Therefore, low levels of vitamin D3 make the endothelial cells of the vessel wall susceptible to oxidative damage due to reduced production of nitric oxide, increased platelet adhesion with the formation of leukocyte micro-aggregates, alteration of the glycocalyx, and increased endothelial inflammation leading to atherothrombosis and ischemia [43].

The role of oxidative stress in BPPV has been demonstrated with increased expression of VCAM-1 and d-ROM, which confirms angiitis’s existence and supports vascular involvement in BPPV [44,45,46]. Furthermore, identifying high levels of d-ROM and VCAM-1 could suggest the usefulness of a drug treatment aimed at correcting oxidative stress and activating endothelial cells [22,23]. Oxidative stress causes endothelial damage and suffering of the macular neuroepithelium and consequent increases in otolithic detachment due to the fragility of the macular surface.

The oral vitamin D supplementation has been associated in our study with ALA, which is considered a “universal antioxidant”, in order to prevent “indirect” oxidative damage caused by calcitriol deficiency [47].

ALA is a natural compound of thiol enzymatically synthesized in the mitochondrion from octanoic acid. The antioxidant properties of ALA include its capacity to scavenge ROS directly and to regenerate endogenous antioxidants, such as glutathione and vitamins E and C, and its metal-chelating activity. Even in its reduced form, dihydrolipoic acid (DHLA) is considered an antioxidant compound [48].

Both ALA and vitamin D3 can cross the blood-brain barrier, and several studies have been conducted to understand their functions within the aging process of the brain. Brain aging has also been related to inflammation and the mitochondrial theory of “free radicals” is one of the most studied hypotheses to explain the molecular mechanism of aging. It is based on the endogenous production of ROS and their damaging effects on mitochondria. ROS modulates cellular mechanisms for cell proliferation, survival and death, and immune responses by inducing the production of proinflammatory factors such as the cytokines that lead to cognitive dysfunction and memory loss [49]. Furthermore, it has been established that excessive secretion of proinflammatory cytokines is a crucial promoter of aberrant inflammatory responses in neurodegenerative diseases [50]. ALA is considered a potential anti-inflammatory agent for neurodegenerative diseases such as Alzheimer’s, showing neuroprotective effects in various neuronal experimental models and modulating various genes involved in cell survival, inflammation, and oxidative stress.

Clinical studies have shown that the administration of ALA associated with vitamin D3 is an effective treatment for treating brain aging by acting on astrocytes in conditions of oxidative stress, with a functional improvement in neurodegenerative diseases, such as Alzheimer’s and Parkinson’s. Therefore, the powerful antioxidant effect of vitamin D3 is greatly enhanced by the association with other antioxidants, such as ALA and curcumin [49,50,51].

ALA and vitamin D3 might exert a synergic effect in modulating inner ear oxidative stress. The results of our study support this hypothesis. In the group of patients treated with associated supplementation of vitamin D and ALA (Arm 1), better results were observed regarding BPPV relapses and subjective perception of symptoms compared to untreated patients or treated with antioxidants only, as demonstrated by the improvement of both DHI and VNS/VAS for post maneuver residual dizziness in Arm 1 patients.

The study has limitations: a central laboratory facility could help to exclude interlaboratory variations, and the measurement of other parameters related to calcium metabolism (calcium, phosphoremia, and alkaline phosphatase) would likely have provided valuable data. Body mass index (BMI) and body fat mass (BFM), correlated with serum 25 (OH) D concentration, were not considered among the study parameters, as well as sun exposure and vitamin D intake with diet, which is difficult to quantify in a large-scale study.

Further studies are therefore desirable to precisely define the relationships between calcium metabolism and vestibular disorders and ratify the need for oral nutritional supplementation targeting these pathways in high recurrence BPPV.

## 5. Conclusions

Patients with frequent relapses of BPPV should be considered at high risk for hypovitaminosis D and routinely screened for this condition following appropriate therapy with repositioning maneuvers.

This study suggested that oral nutritional supplementation with a compound of vitamin D3, ALA, Carnosine, and Zinc (LICA^®^), vitamins of group B, can prevent relapses and reduce symptoms related to high-recurrence BPPV in patients showing insufficient or deficient serum concentration of 25 (OH) D.

The administration of vitamin D in association with a powerful antioxidant (LICA^®^) enhances its effects resulting in reducing the risk of developing new episodes of BPPV and improving its clinical course after maneuvers.

## Figures and Tables

**Table 1:** Demographic data.

		Arm 1 (Vitamin D3 + LICA^®^)	Arm 2 (No Treatment)	Arm 3 (LICA^®^)	*p*-Value
N		93	16	19	
Age		59.7 ± 14.0	62.0 ± 9.4	62.7 ± 12.6	0.6517
Sex	M	62 (66.7%)	13 (81.3%)	14 (73.7%)	0.4602
	F	31 (33.3%)	3 (18.8%)	5 (26.3%)	
Serum 25 (OH) D (ng/mL)		18.2 ± 6.6	36.9 ± 5.7		<0.0001
BPPV recurrences		3.0 ± 4.6	2.5 ± 0.9	2.5 ± 0.9	
Osteoporosis	No	85 (91.4%)	11 (68.8%)	15 (83.3%)	0.0288
	Yes	8 (8.6%)	5 (31.3%)	3 (16.7%)	
DHI		23.5 ± 20.5	32.0 ± 21.3	28.4 ± 21.3	0.137
VAS dizziness		3.6 ± 2.3	3.0 ± 1.9	2.5 ± 1.7	0.1427
VAS vertigo		3.4 ± 2.8	2.9 ± 2.0	3.3 ± 3.0	0.9443
VNS dizziness		3.8 ± 2.4	3.6 ± 2.1	3.2 ± 2.4	0.6383
VNS vertigo		3.7 ± 3.1	2.9 ± 2.1	3.5 ± 3.1	0.8412

**Table 2:** Composition of the study population in absolute numbers and percentages of patients at the time of randomization, during the two follow-up visits, and at the final evaluation. The table shows the patients who dropped out of the study from time to time and the treatment switches. The colorimetric legend indicates the patients who have been reassigned: in green are the subjects whose 25 (OH) D values have gone from “insufficient” or “deficient” to “sufficient” and who switched into Arms 2 and 3; in red, subjects who switched in Arm 1 due to the lowering of the serum 25 (OH) D concentration (below the “sufficiency” threshold). FU: Follow up visit.

Timing	Arm Switch		Arm 1 (Vitamin D3 + LICA^®^)	Arm 2 (No Treatment)	Arm 3 (LICA^®^)	Dropouts
T1 Randomization (n = 128)			93	16	19	
T2 1° FU (n = 109)			83 (76.1%)	10 (9.2%)	16 (14.7%)	19
	Arm 1	74 (67.88%)	70 (84.33%)	0	4 (25%)	
	Arm 2	13 (11.92%)	3 (3.61%)	10 (100 %)	0	
	Arm 3	22 (20.18%)	10 (12.04%)	0	12 (75%)	
T3 2° FU (n = 96)			67 (69.79%)	11 (11.45%)	18 (18.75%)	13
	Arm 1	64 (66.66%)	58 (86.56%)	3 (27.27%)	3 (16.66%)	
	Arm 2	12 (12.5%)	4 (5.97 %)	8 (72.72%)	0	
	Arm 3	20 (20.83%)	5 (7.46%)	0	15 (83.33%)	
T4 Final Visit (n = 89)			60 (67.41%)	12 (13.48%)	17 (19.1%)	7

**Table 3:** Outcomes of ITT multivariate mixed model analysis. T1: randomization visit; T4: end of the study.

		Arm1			Arm2			Arm 3			
	T	Mean (95% CI)	Difference T0–T4 (95% CI)	Within- Group *p*-Value	Mean (95% CI)	Difference T0–T4 (95% CI)	Within- Group *p*-Value	Mean (95% CI)	Difference T0–T4 (95% CI)	Within- Group *p*-Value	Between- Group *p*-Value
**BPPV relapses**	T0	2.76 (1.85–4.12)			2.11 (0.93–4.78)			2.05 (0.9–4.65)			
	T4	0.44 (0.21–0.92)	−2.32 (−3.4–−1.62)	**<0.0001**	0.57 (0.13–2.49)	−1.54 (−3.96–0.34)	0.1113	0.74 (0.2–2.71)	−1.31 (−3.63–0.48)	0.1549	0.9671
**DHI** **score**	T0	30.13 (25.06–35.2)			31.79 (21.04–42.55)			29.08 (18.35–39.81)			
	T4	16.37 (10.67–22.07)	−13.76 (−20.05–−7.47)	**<0.0001**	15.57 (4.41–26.72)	−16.22 (−30.63–−1.82)	0.0278	15.64 (5.2–26.09)	−13.44 (−26.67–0.21)	0.0466	0.9616
**VAS** **dizziness**	T0	3.95 (3.24–4.8)			2.93 (1.89–4.52)			2.47 (1.54–3.98)			
	T6	1.28 (0.89–1.84)	−2.67 (−3.19–−1.81)	**<0.0001**	1.42 (0.71–2.83)	−1.51 (−3–0.17)	0.0719	2.18 (1.31–3.63)	−0.29 (−1.77–1.16)	0.6871	0.8671
**VAS positional vertigo**	T0	3.63 (4.76–37.64)			3.26 (5.72–26.04)			3.95 (7.04–51.87)			
	T6	0.99 (1.54–2.7)	−2.64 (−3.31–−1.64)	**<0.0001**	1.05	−2.21 (−3.77–−0.1)	0.027	2.4	−1.55 (−3.1–0.56)	0.2049	0.614
**VNS** **dizziness**	T0	3.95 (3.25–4.8)			(2.48–2.86)			(4.51–11.07)			
	T6	1.18 (0.89–1.57)	−2.77 (−3.26–−2.04)	**<0.0001**	3.24 (2.16–4.85)	−1.44 (−2.88–0.03)	0.0501	2.82 (1.84–4.31)	−1.14 (−2.6–0.17)	0.0739	0.8231
**VNS positional vertigo**	T0	4.06 (3.13–5.28)			1.8 (1.1–2.93)			1.68 (1.04–2.71)			
	T6	0.8 (0.5–1.29)	−3.26 (−3.81–−2.2)	**<0.0001**	3.39 (1.94–5.95)	−2.06	0.05	4.45 (2.46–8.03)	−3.19 (−4.41–0.63)	0.0099	0.6533

## Data Availability

Not applicable.

## References

[1] Bhattacharyya N., Gubbels S.P., Schwartz S.R., Edlow J.A., El-Kashlan H., Fife T., Holmberg J.M., Mahoney K., Hollingsworth D.B., Roberts R. (2017). Clinical Practice Guideline: Benign Paroxysmal Positional Vertigo (Update). Otolaryngol.-Head Neck Surg..

[2] Imai T., Takeda N., Ikezono T., Shigeno K., Asai M., Watanabe Y., Suzuki M. (2017). Classification, diagnostic criteria and management of benign paroxysmal positional vertigo. Auris Nasus Larynx.

[3] Epley J.M. (1995). Positional vertigo related to semicircular canalithiasis. Otolaryngol.-Head Neck Surg..

[4] Epley J.M. (2001). Human Experience with Canalith Repositioning Maneuvers. Ann. N. Y. Acad. Sci..

[5] Nuti D., Zee D.S., Mandalà M. (2020). Benign Paroxysmal Positional Vertigo: What We Do and Do Not Know. Semin. Neurol..

[6] Von Brevern M., Radtke A., Lezius F., Feldmann M., Ziese T., Lempert T., Neuhauser H. (2007). Epidemiology of benign paroxysmal positional vertigo: A population based study. J. Neurol. Neurosurg. Psychiatry.

[7] von Brevern M., Bertholon P., Brandt T., Fife T., Imai T., Nuti D., Newman-Toker D. (2015). Benign paroxysmal positional vertigo: Diagnostic criteria. J. Vestib. Res..

[8] Libonati G.A., Martellucci S., Castellucci A., Malara P. (2022). Minimum Stimulus Strategy: A step-by-step diagnostic approach to BPPV. J. Neurol. Sci..

[9] Eggers S.D., Bisdorff A., von Brevern M., Zee D.S., Kim J.-S., Perez-Fernandez N., Welgampola M.S., Della Santina C.C., Newman-Toker D.E. (2019). Classification of vestibular signs and examination techniques: Nystagmus and nystagmus-like movements. J. Vestib. Res..

[10] D’Elia A., Quaranta N., Libonati G.A., Ralli G., Morelli A., Inchingolo F., Cialdella F., Martellucci S., Barbara F. (2020). The cochleo-vestibular secretory senescence. J. Gerontol. Geriatr..

[11] Zucca G., Valli S., Valli P., Perin P., Mira E. (1998). Why do benign paroxysmal positional vertigo episodes recover spontaneously?. J. Vestib. Res..

[12] Büki B., Ecker M., Jünger H., Lundberg Y.W. (2013). Vitamin D deficiency and benign paroxysmal positioning vertigo. Med. Hypotheses.

[13] Sheikhzadeh M., Lotfi Y., Mousavi A., Heidari B., Bakhshi E. (2016). The effect of serum vitamin D normalization in preventing recurrences of benign paroxysmal positional vertigo: A case-control study. Casp. J. Intern. Med..

[14] Taneja M.K., Taneja V. (2013). Vitamin D Deficiency in E.N.T. Patients. Indian J. Otolaryngol. Head Neck Surg..

[15] Rhim G.I. (2016). Serum vitamin D and recurrent benign paroxysmal positional vertigo. Laryngoscope Investig. Otolaryngol..

[16] Talaat H.S., Abuhadied G., Talaat A.S., Abdelaal M.S.S. (2015). Low bone mineral density and vitamin D deficiency in patients with benign positional paroxysmal vertigo. Eur. Arch. Oto-Rhino-Laryngol..

[17] Elmoursy M.M., Abbas A.S. (2021). The role of low levels of vitamin D as a co-factor in the relapse of benign paroxysmal positional vertigo (BPPV). Am. J. Otolaryngol..

[18] Pecci R., Mandalà M., Marcari A., Bertolai R., Vannucchi P., Santimone R., Bentivegna L., Di Giustino F., Mengucci A., Vanni S. (2022). Vitamin D Insufficiency/Deficiency in Patients with Recurrent Benign Paroxysmal Positional Vertigo. J. Int. Adv. Otol..

[19] Sheikhzadeh M., Lotfi Y., Mousavi A., Heidari B., Monadi M., Bakhshi E. (2016). Influence of supplemental vitamin D on intensity of benign paroxysmal positional vertigo: A longitudinal clinical study. Casp. J. Intern. Med..

[20] Jeong S.-H., Kim J.-S., Kim H.-J., Choi J.-Y., Koo J.-W., Choi K.-D., Park J.-Y., Lee S.-H., Choi S.-Y., Oh S.-Y. (2020). Prevention of benign paroxysmal positional vertigo with vitamin D supplementation: A randomized trial. Neurology.

[21] Jeong S.-H., Lee S.-U., Kim J.-S. (2022). Prevention of recurrent benign paroxysmal positional vertigo with vitamin D supplementation: A meta-analysis. J. Neurol..

[22] Gucluturk M.T., Unal Z.N., Ismi O., Cimen M.B.Y., Unal M. (2016). The Role of Oxidative Stress and Inflammatory Mediators in Benign Paroxysmal Positional Vertigo. J. Int. Adv. Otol..

[23] Goto F., Hayashi K., Kunihiro T., Ogawa K. (2010). The possible contribution of angiitis to the onset of benign paroxysmal positional vertigo (BPPV). Int. Tinnitus J..

[24] Malara P., Castellucci A., Martellucci S. (2020). Upright head roll test: A new contribution for the diagnosis of lateral semicircular canal benign paroxysmal positional vertigo. Audiol. Res..

[25] Martellucci S., Castellucci A., Malara P., Ralli G., Pagliuca G., Botti C., Gallo A., Ghidini A., Libonati G.A. (2022). Is it possible to diagnose Posterior Semicircular Canal BPPV from the sitting position? The role of the Head Pitch Test and the upright tests along the RALP and LARP planes. Am. J. Otolaryngol..

[26] Martellucci S., Malara P., Castellucci A., Pecci R., Giannoni B., Marcelli V., Scarpa A., Cassandro E., Quaglieri S., Manfrin M.L. (2020). Upright BPPV Protocol: Feasibility of a New Diagnostic Paradigm for Lateral Semicircular Canal Benign Paroxysmal Positional Vertigo Compared to Standard Diagnostic Maneuvers. Front. Neurol..

[27] Nola G., Mostardini C., Salvi C., Ercolani A., Ralli G. (2010). Validity of Italian adaptation of the Dizziness Handicap Inventory (DHI) and evaluation of the quality of life in patients with acute dizziness. Acta Otorhinolaryngol. Ital..

[28] Viprey M., Merle B., Riche B., Freyssenge J., Rippert P., Chakir M.-A., Thomas T., Malochet-Guinamand S., Cortet B., Breuil V. (2021). Development and Validation of a Predictive Model of Hypovitaminosis D in General Adult Population: SCOPYD Study. Nutrients.

[29] Giustina A., Bouillon R., Binkley N., Sempos C., Adler R.A., Bollerslev J., Dawson-Hughes B., Ebeling P.R., Feldman D., Heijboer A. (2020). Controversies in Vitamin D: A Statement From the Third International Conference. JBMR Plus.

[30] Lips P. (2010). Worldwide status of vitamin D nutrition. J. Steroid Biochem. Mol. Biol..

[31] Prentice A., Goldberg G.R., Schoenmakers I. (2008). Vitamin D across the lifecycle: Physiology and biomarkers. Am. J. Clin. Nutr..

[32] Isaia G., Giorgino R., Rini G.B., Bevilacqua M., Maugeri D., Adami S. (2003). Prevalence of hypovitaminosis D in elderly women in Italy: Clinical consequences and risk factors. Osteoporos. Int..

[33] Goldschagg N., Teupser D., Feil K., Strupp M. (2021). No evidence for a specific vitamin D deficit in benign paroxysmal positional vertigo. Eur. J. Neurol..

[34] Melis A., Rizzo D., Gallus R., Leo M.E., Turra N., Masnaghetti D., De Luca L.M., Piras A., Bussu F. (2020). Relationship between calcium metabolism and benign paroxysmal positional vertigo in north Sardinia population. J. Vestib. Res..

[35] Ross M.D. (1979). Calcium Ion Uptake and Exchange in Otoconia. Adv. Otorhinolaryngol..

[36] Thalmann R., Ignatova E., Kachar B., Ornitz D.M., Thalmann I. (2001). Development and Maintenance of Otoconia: Biochemical considerations. Ann. N. Y. Acad. Sci..

[37] Yamauchi D., Nakaya K., Raveendran N.N., Harbidge D.G., Singh R., Wangemann P., Marcus D.C. (2010). Expression of epithelial calcium transport system in rat cochlea and vestibular labyrinth. BMC Physiol..

[38] Yamauchi D., Raveendran N.N., Pondugula S.R., Kampalli S.B., Sanneman J.D., Harbidge D.G., Marcus D.C. (2005). Vitamin D upregulates expression of ECaC1 mRNA in semicircular canal. Biochem. Biophys. Res. Commun..

[39] Vibert D., Sans A., Kompis M., Travo C., Mühlbauer R.C., Tschudi I., Boukhaddaoui H., Häusler R. (2008). Ultrastructural Changes in Otoconia of Osteoporotic Rats. Audiol. Neurotol..

[40] Zhang S., Xing J., Gong Y., Li P., Wang B., Xu L. (2021). Downregulation of VDR in benign paroxysmal positional vertigo patients inhibits otolith-associated protein expression levels. Mol. Med. Rep..

[41] Bigelow R.T., Carey J.P. (2020). Randomized controlled trial in support of vitamin D and calcium supplementation for BPPV. Neurology.

[42] Yang Z., Li J., Zhu Z., He J., Wei X., Xie M. (2021). Effect of vitamin D supplementation on benign paroxysmal positional vertigo recurrence: A meta-analysis. Sci. Prog..

[43] Wee C.L., Mokhtar S.S., Singh K.K.B., Yahaya S., Leung S.W.S., Rasool A.H.G. (2021). Calcitriol Supplementation Ameliorates Microvascular Endothelial Dysfunction in Vitamin D-Deficient Diabetic Rats by Upregulating the Vascular eNOS Protein Expression and Reducing Oxidative Stress. Oxidative Med. Cell. Longev..

[44] Sahin E., Deveci I., Dinc M.E., Ozker B.Y., Bicer C., Erel O. (2018). Oxidative Status in Patients with Benign Paroxysmal Positional Vertigo. J. Int. Adv. Otol..

[45] Li J., Wu R., Xia B., Wang X., Xue M. (2020). Serum levels of superoxide dismutases in patients with benign paroxysmal positional vertigo. Biosci. Rep..

[46] Xie K.-H., Liu L.-L., Su C.-Y., Huang X.-F., Wu B.-X., Liu R.-N., Li H., Chen Q.-Q., He J.-S., Ruan Y.-K. (2020). Low Antioxidant Status of Serum Uric Acid, Bilirubin, Albumin, and Creatinine in Patients with Benign Paroxysmal Positional Vertigo. Front. Neurol..

[47] Ajith T.A. (2020). Alpha-lipoic acid: A possible pharmacological agent for treating dry eye disease and retinopathy in diabetes. Clin. Exp. Pharmacol. Physiol..

[48] DeRosa G., D’Angelo A., Romano D., Maffioli P. (2016). A Clinical Trial about a Food Supplement Containing α-Lipoic Acid on Oxidative Stress Markers in Type 2 Diabetic Patients. Int. J. Mol. Sci..

[49] Molinari C., Morsanuto V., Ghirlanda S., Ruga S., Notte F., Gaetano L., Uberti F. (2019). Role of Combined Lipoic Acid and Vitamin D3 on Astrocytes as a Way to Prevent Brain Ageing by Induced Oxidative Stress and Iron Accumulation. Oxidative Med. Cell. Longev..

[50] Farghali M., Ruga S., Morsanuto V., Uberti F. (2020). Can Brain Health Be Supported by Vitamin D-Based Supplements? A Critical Review. Brain Sci..

[51] Alamro A.A., Alsulami E.A., Almutlaq M., Alghamedi A., Alokail M., Haq S.H. (2020). Therapeutic Potential of Vitamin D and Curcumin in an In Vitro Model of Alzheimer Disease. J. Cent. Nerv. Syst. Dis..

